# Which illicit drugs are injected in Oslo? A study based on analysis of drug residues in used injection equipment and self-reported information

**DOI:** 10.1177/14034948211043984

**Published:** 2021-09-18

**Authors:** Hallvard Gjerde, Anne Line Bretteville-Jensen, Lihn Bache-Andreassen, Kristin Hanoa, Håvard Furuhaugen, Gerd-Wenche Brochmann, Vigdis Vindenes

**Affiliations:** 1 Section of Drug Abuse Research, Department of Forensic Sciences, Oslo University Hospital, Norway; 2 Department of Alcohol, Tobacco and Drugs, Norwegian Institute of Public Health, Norway; 3 Institute of Clinical Medicine, Faculty of Medicine, University of Oslo, Norway

**Keywords:** Illicit drugs, people who inject drugs, injecting paraphernalia, used syringes, needle exchange, chemical analysis, liquid chromatography–mass spectrometry, survey

## Abstract

**Background:**

People who inject drugs (PWID) have a high risk of premature death due to fatal overdoses. Newly emerged fentanyls, much more potent than heroin and other opioids, may increase this risk further. Therefore, precise information on injected drugs is critical to improving prevention strategies.

**Aims:**

This study aimed to analyse drug residues in used injection equipment in order to determine drug and drug combinations and compare and complement findings with self-reported information.

**Methods:**

Used syringes and needles (*n*=766) were collected at the supervised drug consumption facilities, the needle exchange service and two low-threshold health services for problem drug users in Oslo, Norway. The material was collected every third month from June 2019 to June 2020 and analysed for 64 substances using highly specific analytical methods (ultra–high performance liquid chromatography tandem mass spectrometry). Additionally, a street-recruited sample of PWID was interviewed from 2017 to 2019 regarding their drug injection habits (*n*=572).

**Results:**

Heroin (65.5%) or amphetamines (59.8%), often in combination (30.5%), were commonly detected in drug residues. Other opioids, stimulants or benzodiazepines were rarely detected (6.1%). Fentanyl was detected in only one syringe. Heroin was the most reported drug (77.6% during the past four weeks, 48.3% daily/almost daily), followed by amphetamines (57.5% during the past four weeks, 23.1% daily or almost daily). Injection of methadone, buprenorphine and dissolved tablets was self-reported more frequently than determined in drug residue findings.

**Conclusions:**

**Analysis of the injection equipment proved useful as a non-invasive, rapid and accurate means to obtain detailed information on injected drugs in Oslo and supplement traditional PWID survey information.**

## Introduction

People who inject drugs (PWID) present a 10–20 times higher mortality rate than the general population. Fatal overdoses are the most common cause of premature death [1]. Causes for overdosing include a lack of accurate information regarding the type and purity of drugs bought on the illicit market, drug users overestimating their drug tolerance and a combined intake of heroin or other opioids with alcohol, benzodiazepines or stimulants. In Norway, overdose deaths account for a higher proportion of deaths among 15–44 years old (13.8%) than the total number of deaths caused by traffic accidents, falls, drowning and homicide (7.5%) [2]. Therefore, reducing the number of drug-induced deaths has long been high on the political agenda. In order to design and implement prevention strategies better, improved knowledge regarding what drugs and drug combinations are being used by PWID is warranted.

Recent developments have further expedited the need for comprehensive knowledge [3]. In addition to the injection of traditional opioids such as heroin and morphine, increased availability of synthetic opioids, such as fentanyl and fentanyl analogues, on the illicit drug market has contributed to a dramatic increase in the number of overdose deaths in the USA, Canada, Sweden and other European states [4]. Fentanyl is a potent analgesic drug used clinically. It is 50–100 times more potent than morphine. As an analgesic medication, fentanyl may be prescribed as a patch placed on the patient’s skin or as lozenges to be placed under the tongue. For acute medical treatment, fentanyl is administered by injection or infusion. On the illegal market, fentanyl may be sold as a powder, dropped onto blotter paper and added to eye droppers and nasal sprays [5]. Fentanyls are also mixed with other drugs sold illegally. For example, heroin may be laced with fentanyls [6], and counterfeit tablets labelled Xanax containing fentanyl instead of alprazolam have been available on the illicit drug market in several countries [7].

Some fentanyl analogues are extremely potent. For example, carfentanil is 10,000 times more potent than morphine, and a small grain (0.02 mg) constitutes a lethal dose [8]. A high number of carfentanil seizures have been reported in the Baltic states (i.e. Estonia, Latvia and Lithuania) [9]. These countries are crucial for Scandinavian trade, tourism and employment immigration, with close ties indicating the potential for the illegal entry of fentanyls from these countries. Carfentanil and other potent fentanyls have already caused overdose deaths in Scandinavia [10,11]. Increased knowledge of the extent and types of fentanyls present on the illegal market, whether sold as such or disguised as other drugs, could reduce overdose risks by PWID when informed, and analysis of drug residues in used syringes and needles can be one strategy to close the information gap.

Typically, information on drug use among PWIDs has been based on interviews with convenience samples of users. However, people might under-report or may not be aware of the actual content of purchased drugs. One example is para-methoxymethamphetamine (PMMA) sold as amphetamine or Ecstasy (3,4-metylendioksymetamphetamine) in Norway in 2010. Approximately 30 people died from PMMA poisoning in a short period [12].

In addition to the risk of premature death, drug injections may cause other harmful health effects. Injections carry the risk of abscesses and blood-borne diseases, such as hepatitis C and human immunodeficiency virus. Interviews with PWID further indicate that injection of dissolved tablets is common, either alone or combined with opioids or stimulants, to increase the desired drug effect [13]. Tablets often contain poorly soluble ingredients. Injecting tablet particles may cause serious harm, including infections and obstruction of small blood vessels, which in turn may lead to acute limb ischaemia (inadequate blood flow) and, in worst cases, amputations. In addition, accumulation in lung tissue has been reported to form granulomas and non-functioning scar tissue or fibrosis [14,15]. Improved information on tablet injection will help design better programmes to reduce health risks among PWIDs.

Norway has one of the highest rates of drug-induced deaths in Europe. The average number of deaths induced by psychoactive drug use was 267 per year during 2009–2019 [16], which corresponds to approximately 75 per million population aged 15–64 years. The average overdose mortality rate in Europe in 2017 was approximately 23 deaths per million people aged 15–64 years [3]. However, the numbers are not necessarily comparable because countries may vary in the extent to which they are able to identify drug-induced deaths correctly and in their definitions of these deaths. Four out of five overdose deaths in Norway were caused by opioids, very often by means of intravenous injection. Autopsies have revealed that most often, the concomitant use of alcohol or benzodiazepines is involved, in addition to opioids [17]. The elevated prevalence of drug-induced deaths emphasises the particular importance of improved and timely information, which would also afford complementary data to self-reports.

Analysis of drug residues in used syringes is a relatively new approach for obtaining objective data on injected drugs. Data collection is non-invasive, and the method has the potential to capture changes in the illicit market for injectable drugs and provide an opportunity to identify new psychoactive substances. The methodology has previously been used in some European countries, the USA and Australia [18–25]. Our study adds to the current literature by (a) presenting analytical results from drug residues collected in Oslo; (b) testing for 64 substances, including 24 fentanyls and six other opioids and (c) not confining analyses to drug residues from used syringe barrels only but also from used needles; and (d) comparing drug test results to self-reported information from PWID.

## Methods

### Collection of used syringes and needles

In the present study, syringes and needles were collected in Oslo in June, September and December 2019 and in March 2020 at the city’s supervised drug consumption facilities and the distribution site for needles and syringes, which are operated by the Oslo city authorities, and at a low-threshold health service for problem drug users operated by a non-governmental organisation. In June 2020, the supervised drug consumption facilities were closed due to the COVID-19 epidemic. Therefore, samples were collected at the syringe distribution site and two low-threshold health services. A minimum of 50 syringes or needles were collected from each location. The collection of the used syringe barrels was prioritised, if available, because the concentrations of residual drugs are higher in used barrels than in the needles and therefore easier to detect and identify.

In previous studies assessing drug residues, only remains found in the used syringe barrels were analysed [18–25]. In Norway, it is substantially more challenging to find used syringe barrels than needles for analysis, as syringes in their entirety (barrels and needles) are returned to distribution points to a lesser extent. Conversely, used needles are often returned. Specially designed small disposal boxes containing up to 23 needles are used for this purpose (see Supplemental Figure S1).

Each collected syringe barrel and one needle from each needle disposal box were selected for drug testing. Trace analysis for 64 drug types was performed using ultra–high performance liquid chromatography tandem mass spectrometric detection, a sensitive and specific analytical technique. The details are described in the Supplemental Material.

### Survey among PWID

Survey data were obtained from interviews with PWID conducted outside the main needle exchange programme (NEP) facility in Oslo city centre in 2017, 2018 and 2019. The interviews were part of an ongoing study that began in 1993. Participants were approached after they had collected the injecting equipment from the NEP facility. Each interview was conducted out of earshot from others and took approximately 15 minutes to complete. Among those who declined to participate, the most common reasons for refusal were that they did not have sufficient time or were experiencing withdrawal symptoms. No names or other personal information were collected. Participants were asked what type of drugs and their frequency of injection in the four weeks leading up to the interview. Detailed information regarding the survey has been previously published [26].

### Ethical assessment

The syringes and needles used were randomly collected from the disposal bins and could not be linked to individuals. The data protection official at Oslo University Hospital was informed regarding the collection and analysis of used syringes and needles. The data protection official at the Norwegian Institute of Public Health was informed about the anonymous survey of drug use among PWID. Anonymous studies, such as the present undertaking, are not regulated by the Health Research Act, the Research Ethics Act or the General Data Protection Regulation.

## Results

### Drug residues

A total of 782 syringes or needles were selected for analysis. It was impossible to analyse 16 of the syringes and needles, either because the needles were clogged or because the extracts contained too much blood that would contaminate the instrument. No drug was found in extracts from 33 used syringes or needles. Thus, drugs were detected in 733 (96%) of the 766 analysed samples (406 syringe barrels and 360 needles). The total drug findings are presented in Table I. Heroin was detected in 65.5% of samples and amphetamines in 59.8%. Other drugs were detected in 6.1% of samples, primarily other opioids (2.2%), benzodiazepines (2.1%) and other stimulants (2.1%).

**Table I.** Comparison of findings in used syringes (2019–2020) with self-reported drug injections (2017–2019).

Drug type	Findings in used syringes, % (*N*=766)	Self-reported drug injections, % (*N*=572)	
		Daily or almost daily	Past four weeks
Heroin	65.5	48.3	77.6
Amphetamine	59.8	23.1	57.5
Morphine	0.5	1.2	9.3
Cocaine	0.7	0.4	5.8
Methadone	0.9	3.5	10.5
Buprenorphine	0.7	3.3	8.2
Tablets^a^	2.1	8.6	27.8
Clonazepam	1.2	–	–
Alprazolam	0.9	–	–
Other	2.2	0.5	6.5
MDMA^b^	1.0	–	–
Methylphenidate	0.3	–	–
Ethylphenidate	0.1	–	–
Fentanyl	0.1	–	–

a Sedatives and hypnotics.

b 3,4-metylendioksymetamphetamine (Ecstasy).

Drug combinations are presented in Table II. The most common combination was heroin and amphetamines, sometimes combined with additional drugs (30.5% in total). We did not detect any regular ratio between amphetamines and heroin. The mixture was therefore most likely decided by individual preferences or by chance, varying from <10% to 50% (w/w) of one drug mixed with the other (results not shown). The second most common combination was heroin and a benzodiazepine (clonazepam or alprazolam), sometimes combined with additional drugs (2.0% in total).

**Table II.** Drug combinations in extracts from 766 used syringes or needles.

Drug combinations	%
Heroin and amphetamines	28.3
Heroin, amphetamines and other drugs^a,b,c^	2.2
Heroin and benzodiazepine^b^	1.0
Heroin and cocaine	0.3
Heroin and other opioids^a^	0.3
Amphetamines and other opioids^a^	0.8
Amphetamines, other opioids^a^ and benzodiazepines^b^	0.1
Amphetamines and other stimulants^c^	0.7

a Buprenorphine, fentanyl, methadone or morphine.

b Alprazolam or clonazepam.

c Ethylphenidate, methylphenidate, MDMA or cocaine.

### Self-reported drug injection

Self-reported information on drug injections performed during the past four weeks prior to the interview was obtained from 572 PWID. Data were compared with drug findings in the injection equipment used, elaborated in Table I. The most commonly reported drug injected was heroin, followed by amphetamine. Approximately half reported daily or almost daily injections of heroin, whereas a quarter reported daily or almost daily injections of amphetamine. Almost a third reported injecting tablets during the last month, but <10% reported injecting tablets daily or almost daily. Few participants reported injections of morphine, cocaine or other drugs.

## Discussion

The analysis of 64 substances, including 24 fentanyls, in drug residues detected that heroin and amphetamines were the most commonly injected drugs in Oslo. Each substance was found in 60–66% of the samples. Few drug users had injected other opioids, other stimulants or benzodiazepines. Fentanyl was detected once, while other highly toxic fentanyls were not detected. Almost a third of the samples indicated combined use of amphetamines and heroin. This is worrying because such combinations may increase the risk of fatal overdose. By combining those drugs, users want to experience an intense rush while hoping to reduce some negative effects of both substances. It might, however, be difficult for the user to tell when an overdose point is approaching. Also, amphetamines may cause arrhythmias, heart failure or stroke. To date, surveys among PWID have not on a regular basis asked about injection of drug mixtures. This should be considered in future studies.

Combining heroin with stimulants is also common in other parts of the world, but more often with cocaine than amphetamines [27]. In Oslo, cocaine was rarely detected in used syringes. The users seemingly preferred injecting amphetamines as stimulants. This finding could be explained by the fact that amphetamines are cheaper and demonstrate longer-lasting effects.

Heroin was also the most commonly reported drug, followed by amphetamines. Despite distinct methodological approaches and a lack of complete overlap in time points and data-collection locations, the concurrence shown in Table I suggests that both self-report and analysis of used injection paraphernalia reflect the same pattern of drug injection in Oslo. Analysis of drug residues complemented survey information by revealing that almost a third had injected mixtures of heroin with amphetamines.

Autopsy results from drug-induced deaths confirm that heroin is often combined with other drugs. For example, a study of 1288 heroin-related overdose deaths in Norway (2000–2017) revealed that 75% of the blood samples tested positive for benzodiazepines, while 33% tested positive for amphetamines [28]. In our study assessing used syringes and needles, 47% that tested positive for heroin were also positive for amphetamines, and 3% tested positive for benzodiazepines. This may suggest that people who combine heroin with other drugs often seem to administer amphetamines by injection and benzodiazepines by oral administration.

Drug residue analysis also complemented the self-reported information regarding the injection of fentanyls. Drug users were not explicitly asked about these opioids and might not have complete information regarding the injected drugs. Accordingly, analysis of drug residues could provide timely information to users and health authorities regarding the local availability of these high-risk drugs. In the present study, fentanyl was detected in one syringe only, in combination with heroin. Heroin is sometimes laced with fentanyl to enhance its potency. However, we lack information to confirm whether this was the reason fentanyl was detected. No other fentanyls were detected. Our findings thus suggest that injecting fentanyl and fentanyl derivatives is a rare event among PWID in Norway.

In Lithuania, 33% of the syringes used in 2019 contained traces of carfentanil, most often combined with methadone but sometimes along with adulterants only, whereas fentanyls were rarely detected in used syringes from other European cities [22]. In a study assessing syringes used in New York City, 17% tested positive for fentanyls [25]. Importantly, however, fentanyls may be administered by alternate routes. Therefore, analysis of fentanyls in used syringes may not reflect the total incidence of use among problem drug users.

Our two data sources regarding drug injection also revealed some interesting differences. The self-reported rates of daily or almost daily injections of morphine, methadone, buprenorphine and crushed tablets were higher than those in used syringes or needles. Analysis of residual drugs found that only alprazolam and clonazepam tablets were crushed, dissolved and injected, corroborating with information in drug user forums on the Internet. These substances are more potent than diazepam and oxazepam. Injecting the most potent benzodiazepines reduces the amount of particulate matter injected, thus reducing the risk of tissue damage. In addition, self-reported data on the types of drugs injected at the supervised drug consumption facilities indicate that benzodiazepines were rarely injected. From April to December 2019, only 2.4% of users reported injection of anxiolytics or tranquillisers [29]. PWID must be encouraged to take tablets orally instead of intravenously because of the dangers associated with injecting crushed and dissolved tablets.

The needles proved valuable for the analytical testing of drug residues. A sufficiently large amount of drug residues could be detected in most cases. No drug traces were found in the 33 syringes or needles used. The most likely reason is that the equipment had been rinsed after use, had not been used or was used to inject substances that were not included in the analytical programme, such as anabolic steroids, other psychoactive substances seldom used by PWID in Oslo or only adulterants.

Findings from other drug residue studies highlight the need to conduct local studies to determine relevant data regarding drug usage for designing targeted prevention programmes. Accumulating evidence has revealed considerably different drug preferences among PWID across examined cities: injections of heroin and cocaine were most common in Glasgow (UK), Cologne (Germany), Lausanne (Switzerland) and New York (USA); injections of heroin and cathinones were most common in Budapest (Hungary) and Paris (France); injection of buprenorphine and amphetamines were most common in Helsinki (Finland); injection of methadone and carfentanil were most common in Vilnius (Lithuania); whereas in Sydney, heroin and amphetamines were most commonly injected, as observed in Oslo [21–24]. Differences between cities may be explained by factors such as drug availability, prices and drug use in cultures in the PWID community, and these need to be reflected in locally adjusted harm reduction and prevention measures.

### Limitations

The syringes and needles used in this study were selected from four sites in Oslo during 2019–2020. Still, the selection may not be representative for all injections of illegal drugs by PWID during the study period. Users who do not visit these collection sites may have other drug preferences, and people may inject different drugs when using the supervised injection room than elsewhere.

Some findings of drug traces might be due to contamination by reuse of syringes, by contact with other used syringes or needles in the containers for used paraphernalia or by the presence of drugs in residual blood in the syringes or needles.

The analytical test results represent only the drugs administered by injection. Most users may have administered other drugs orally.

Data on self-reported drug injections were collected over a longer time frame (2017–2019) close to the NEP facility and the supervised drug consumption facilities and may therefore not represent the same cohorts as those who delivered used syringes. Also, drug use surveys may not provide accurate data because of recall bias, under-reporting or incorrect information about purchased drugs. The users did not report the injection of drug mixtures or only reported the main substance.

## Conclusions

Analysis of drug residues in used syringes and needles is a non-invasive, rapid and accurate means to obtain detailed and timely information regarding the types of substances injected and their relative frequency of use. Thus, they could supplement traditional survey information. The vast majority of PWID in Oslo seems to inject heroin or amphetamines, often in combination, whereas other opioids (including fentanyls), other stimulants or benzodiazepines may be injected more rarely.

## Supplemental Material

sj-docx-1-sjp-10.1177_14034948211043984 – Supplemental material for Which illicit drugs are injected in Oslo? A study based on analysis of drug residues in used injection equipment and self-reported information Click here for additional data file. Supplemental material, sj-docx-1-sjp-10.1177_14034948211043984 for Which illicit drugs are injected in Oslo? A study based on analysis of drug residues in used injection equipment and self-reported information by Hallvard Gjerde, Anne Line Bretteville-Jensen, Lihn Bache-Andreassen, Kristin Hanoa, Håvard Furuhaugen, Gerd-Wenche Brochmann and Vigdis Vindenes in Scandinavian Journal of Public Health
