# Supplementary material for: Which illicit drugs are injected in Oslo? A study based on analysis of drug residues in used injection equipment and self-reported information
Source: Scand J Public Health. 2021 Sep 18;51(1):21–7. doi: 10.1177/14034948211043984 (PMC9900188; doi:10.1177/14034948211043984)
Supplement: sj-docx-1-sjp-10.1177_14034948211043984 – Supplemental material for Which illicit drugs are injected in Oslo? A study based on analysis of drug residues in used injection equipment and self-reported information [file sj-docx-1-sjp-10.1177_14034948211043984.docx]

**Supplemental Material**


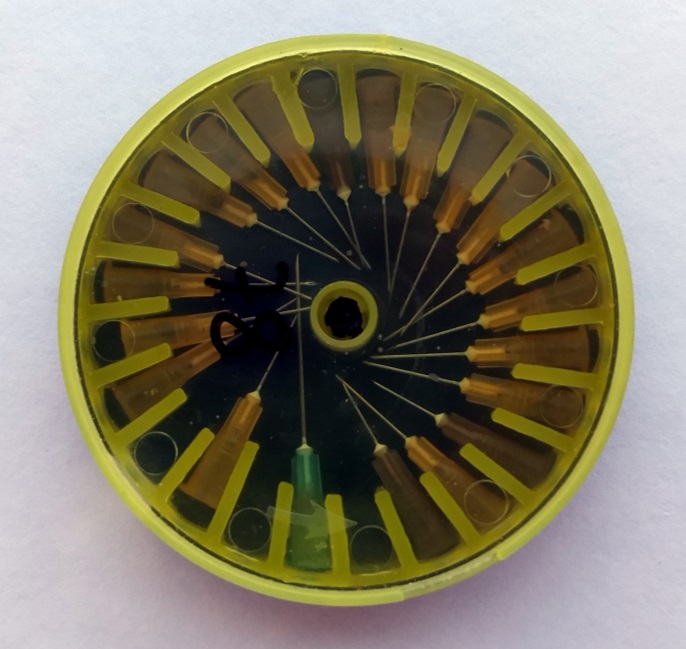


**Figure S1.** Small disposal box for 23 used needles.

**Preparation and analysis of residual drugs in syringes and needles**

Each syringe or needle was washed a minimum of five times with 1 mL of methanol. Needles clogged by dried or coagulated blood were rinsed and shaken with 1 mL of methanol for a minimum of 15 min. Particles were removed from the extracts using 0.2 μm filters (Whatman™ Mini-UniPrep™ Syringeless PTFE filter, GE Healthcare Bio-Sciences, Pittsburgh, PA, USA). The filtered solutions were diluted with water (1:10 for analysis of fentanyls and 1:100 for the analysis of other substances).

Quantitative analysis of 64 drugs and 2 degradation products (Table S1) was performed using ultra-high-performance liquid chromatography (UHPLC) with tandem mass spectrometric (MS/MS) detection and external standard calibration. Previously published methods were used, with slight modifications [S1-S2]; see Table S2 for details.

The total amounts (ng) of the detected drugs listed in Table S1 were calculated. In order to exclude results that were likely due to contamination, findings of drugs below 5% (w/w) of the total amount of detected drugs were disregarded, except for buprenorphine and the most potent benzodiazepines (alprazolam, clonazepam, diclazepam, etizolam, flubromazolam, flunitrazepam, phenazepam) where a limit of 2.5% (w/w) was used. For fentanyls, all findings above the listed cut-off values were reported.

**Table S1.** Psychoactive substances included in analytical testing and their cut-off concentrations.

| **Substance group** | **Substance** | **Cut-off (µg)** |
| --- | --- | --- |
| Amphetamines^a^ | Amphetamine | 0.3 |
|  | Methamphetamine | 0.3 |
| Cocaine^a^ | Cocaine | 0.6 |
|  | Benzoylecgonine (from cocaine) | 0.6 |
| Heroin | Heroin | 0.4 |
|  | 6-monoacetylmorfin (from heroin) | 1.3 |
| Other opioids, including fentanyls | Buprenorphine | 0.4 |
|  | Codeine | 0.6 |
|  | Methadone | 0.6 |
|  | Morphine | 0.6 |
|  | Tramadol | 0.5 |
|  | 2-fluorfentanyl | 0.003 |
|  | 3-fluorfentanyl | 0.003 |
|  | 4-fluorfentanyl | 0.003 |
|  | 4-chlorisobutyrfentanyl | 0.003 |
|  | 4-fluorbutyrfentanyl | 0.003 |
|  | 4-fluorisobutyrfentanyl | 0.003 |
|  | 4-methoxy-butyrfentanyl | 0.003 |
|  | Acetylfentanyl | 0.003 |
|  | Acetylnorfentanyl | 0.002 |
|  | Acrylfentanyl | 0.003 |
|  | Alfentanil | 0.003 |
|  | Benzodioxolefentanyl | 0.003 |
|  | Butyrfentanyl | 0.003 |
|  | Carfentanil | 0.003 |
|  | Cis-3-methylfentanyl | 0.003 |
|  | Cyclopropylfentanyl | 0.003 |
|  | Despropionyl-2-fluorfentanyl | 0.002 |
|  | Fentanyl | 0.2 |
|  | Furanylfentanyl | 0.003 |
|  | Metoxyacetylfentanyl | 0.003 |
|  | Norcarfentanil | 0.003 |
|  | Norfentanyl | 0.002 |
|  | Sufentanil | 0.003 |
|  | Valerylfentanyl | 0.003 |
| Anxiolytics, tranquillisers, hypnotics | Alprazolam | 0.6 |
|  | Clonazepam | 0.5 |
|  | Diazepam | 0.6 |
|  | Diclazepam | 0.6 |
|  | Etizolam | 0.009 |
|  | Flubromazepam | 0.009 |
|  | Flubromazolam | 0.009 |
|  | Flunitrazepam | 0.6 |
|  | Midazolam | 0.03 |
|  | Nitrazepam | 0.6 |
|  | Oxazepam | 1.4 |
|  | Phenazepam | 0.009 |
|  | Zolpidem | 2.5 |
|  | Zopiclone | 3.1 |

(continues)

**Table S1 (continued).** Psychoactive substances included in analytical testing and cut-off concentrations.

| **Substance group** | **Substance** | **Cut-off (µg)** |
| --- | --- | --- |
| Cathinones^a^ | 3-MMC | 0.004 |
|  | 4-MEC | 0.005 |
|  | Alpha-PVP | 0.006 |
|  | MDPV | 0.007 |
|  | Mephedrone (4-MMC) | 0.004 |
|  | Methylone | 0.02 |
|  | Pentedrone | 0.01 |
| Tryptamines^b^ | Dimethyltryptamine | 0.005 |
| Phenethylamines^a^ | 2C-B | 0.003 |
|  | 2C-I | 0.003 |
|  | 25I-NB20Me | 0.004 |
|  | 25C-NB20Me | 0.003 |
|  | MDMA (Ecstasy) | 0.4 |
| Piperidines^a,b^ | Ethylphenidate | 0.006 |
|  | Methylphenidate | 0.006 |
| Cyclohexanones^c^ | Ketamine | 0.002 |
| Thiophene stimulants^a^ | Methiopropamine^a^ | 0.004 |

^a^Psychostimulants. ^b^Psychedelics/hallucinogenics. ^c^Dissociatives.

**Table S2:** Analytical conditions for ultra-high performance liquid chromatography with tandem mass spectrometric analysis

|  | **Fentanyls** | **Other drugs** |
| --- | --- | --- |
| UHPLC instrument | Waters Acquity | Agilent 1290 Infinity |
| Column | Phenomenex Kinetex Biphenyl (100 mm, 2.1 mm i.d., 1.7 µm) | Waters Acquity HSS T3  (100 mm, 2.1 mm i.d., 1.8 µm) |
| Eluation | Eluent A: 10 mM formate buffer, pH 3.1. Eluent B: 100 % methanol.  0.6 ml/min, gradient.  Run time: 9 min. | Eluent A: 10mM formate buffer, pH 3.1.  Eluent B: 100 % methanol.  0.5 ml/min, gradient.  Run time: 9.1 min |
| Temperature | 60°C | 65°C |
| MS/MS instrument | Wates Xevo-TQS Triple Quadrupole | Agilent 6490 Triple Quadrupole |
| Temperature | Ion source: 150 °C.  Desolvation: 500 °C | Ion source: 250 °C |
| Scan | Multiple reaction monitoring (MRM). Positive electrospray (ESI+). | Dynamic multiple reaction monitoring (dMRM). Positive electrospray (ESI+) |

# References

[S1] Bergh, M. S., Bogen, I. L., Wilson, S. R., & Øiestad, A. M. L. (2018). Addressing the fentanyl analogue epidemic by multiplex UHPLC-MS/MS analysis of whole blood. Therapeutic Drug Monitoring, 40(6), 738-748. <https://doi.org/10.1097/ftd.0000000000000564>

[S2] Valen, A., Øiestad, A. M., Strand, D. H., Skari, R., & Berg, T. (2017). Determination of 21 drugs in oral fluid using fully automated supported liquid extraction and UHPLC-MS/MS. Drug Testing and Analysis, 9(5), 808-823. <https://doi.org/10.1002/dta.2045>
